# The Probiotic Strain *Bifidobacterium animalis* ssp. *lactis* HY8002 Potentially Improves the Mucosal Integrity of an Altered Intestinal Microbial Environment

**DOI:** 10.3389/fmicb.2022.817591

**Published:** 2022-04-29

**Authors:** Joo Yun Kim, So-Jung Bang, Ju-Yeon Kim, Eun Jung Choi, Keon Heo, Jae-Jung Shim, Jung-Lyoul Lee

**Affiliations:** R & BD Center, hy Co., Ltd., Yongin-si, South Korea

**Keywords:** intestinal microbiome, Peyer’s patches, IgA, tight junctions, dysbiosis, antibiotics—immune effect

## Abstract

Intestinal microbiota mediate the development and regulation of the intestinal immune system either directly or indirectly. Particularly, *Bifidobacterium* spp. play an important role in regulating the intestinal immunity and intestinal barrier. We demonstrated that *Bifidobacterium animalis* ssp. *lactis* HY8002, selected from eight *Bifidobacterium* strains by *in vitro* experimentation, had exceptional resistance to digestive tract conditions and high adhesion to intestinal epithelial cells and a positive effect on immunoglobulin A (IgA) secretion by Peyer’s patch cells. Moreover, HY8002 restored the expression of tight junction-related genes, initially reduced by lipopolysaccharide treatment, to normal levels in human intestinal epithelial cells. Notably, HY8002 restored kanamycin-induced reduction in Peyer’s patch cell numbers, serum and fecal IgA levels, and zonula occludens 1 and Toll-like receptor 2 levels in the mouse small intestine. In addition, HY8002 restores microbiome composition disturbed by kanamycin, and these microbiome changes have been found to correlate with TLR2 levels in the small intestine. Moreover, the ability of HY8002 to enhance IgA in Peyer’s patch cells and ZO-1 levels in intestinal epithelial cells was significantly inhibited by a TLR2 blocking antibody, which suggests that the HY8002 improve intestinal barrier function *via* TLR2. Finally, whole-genome sequencing of HY8002 revealed that it did not possess any known virulence factors. Therefore, HY8002 is a promising, functional probiotic supplement to improve intestinal barrier function by improving intestinal immunity and microbiota balance.

## Introduction

The mucosal immune system protects the body from foreign substances, such as pathogens and food allergens, and is closely associated with homeostasis (Brandtzaeg et al., 1999; Holmgren and Czerkinsky, 2005; Levit et al., 2017). In particular, mucosal immunity plays a crucial role in protecting intestinal mucosa, which has a large surface area that is exposed to the external environment. Therefore, the mucosal-associated lymphoid tissue (MALT) forms a large area of the intestinal lymphoid tissue and consists of Peyer’s patches, lamina propria, and mesenteric lymph nodes (Hansen and Sams, 2018; Park et al., 2020). Peyer’s patches can be induced to secrete immunoglobulin A (IgA) to the mucosal surface; they play the most important role as an intestinal immune barrier (Fagarasan and Honjo, 2003; Cerutti and Rescigno, 2008; Macpherson et al., 2008; Hara et al., 2019). The IgA immunoglobulin accounts for nearly 80% of all the antibodies produced in the mucosal tissues and prevents the absorption of antigens at mucosal surfaces by attaching to bacteria or viruses (Macpherson et al., 2000; Cerutti and Rescigno, 2008; Kim et al., 2016). It is the first line of defense and ensures both immune exclusion and neutralization of the translocated bacteria. Thus, it is an important regulator of bacteria-induced inflammation and preserves the integrity of the intestinal barrier (Cunningham-Rundles, 2001; Macpherson et al., 2001; Boullier et al., 2009; Park et al., 2020). Intercellular movement of molecules in the intestine is regulated by complex interactions among numerous proteins in tight junctions, which connect intestinal epithelial cells. Therefore, tight junctions play an important role in maintaining the integrity of the intestinal epithelial barrier (Suzuki, 2013; Feng et al., 2019). Proteins, such as zona occludens 1 (ZO-1) and occludin (OCLN), that constitute tight junctions are selectively regulated by protein kinase C and have also been reported to be associated with Toll-like receptors (TLRs; Stuart and Nigam, 1995; Cario et al., 2004; Gu et al., 2016). The TLR family of receptors regulate the immune system by recognizing and discriminating between foreign pathogens and endogenous molecules. Previously, TLRs were known to be involved in innate immunity alone; however, studies have found that they play a key role in linking innate and acquired immunities. While 10 functional TLRs have been discovered in humans, research on each of their function and any related diseases is ongoing (El-Zayat et al., 2019).

*Bifidobacterium* spp. are gut microbes that play an important role in promoting a favorable intestinal ecosystem, and they exhibit immunomodulatory effects in both animals and humans (Hill et al., 2017; Shang et al., 2020). Studies have found that some *Bifidobacterium* spp. coexist beneficially with commensal microbes in the gut and have positive effects on the immune system (Routy et al., 2018; Bonfrate et al., 2020; Shang et al., 2020). These positive effects are attributable to interactions between specific molecules expressed by the *Bifidobacterium* spp. and pattern recognition receptors, such as TLRs, present on intestinal epithelial and immune cells (Meng et al., 2016; Ruiz et al., 2017; Shang et al., 2020). However, not all *Bifidobacterium* spp. exhibit identical immunoregulatory activities; rather, various strains of a single species may exhibit different immunoregulatory characteristics (Medina et al., 2007; Menard et al., 2008; Ruiz et al., 2017).

Broad-spectrum antibiotics promote intestinal bacterial imbalance, called gut dysbiosis, by dramatically reducing the diversity and taxonomic richness of the gut microbiota (Antonopoulos et al., 2009; Rea et al., 2011; Liu et al., 2020). Gut dysbiosis impairs the integrity of the intestinal barrier by inhibiting tight junction-related protein synthesis in intestinal epithelial cells and IgA secretion in Peyer’s patches (Maruya et al., 2013; Kim et al., 2016; Feng et al., 2019). Disrupting this integrity can ultimately lead to and exacerbate gastrointestinal diseases and systemic immune and metabolic disorders (Carding et al., 2015; Feng et al., 2019; Ferreira et al., 2020). Of note, probiotics and prebiotics are being proposed as emerging dietary supplements that can prevent and improve gut dysbiosis by regulating the composition of the gut microbiota (Lee et al., 2006; Liu et al., 2020; Shi et al., 2020).

Several studies have attempted to use *Bifidobacterium* spp. to prevent and alleviate intestinal dysbiosis and dysbiosis-associated diseases (Medina et al., 2007; Meng et al., 2016; Lee et al., 2019; Invernici et al., 2020). However, most of these studies have only validated the mechanism of action and disease improvement properties of the bifidobacterial strains. They have not evaluated the characteristics or the potential of the strains as probiotics. Therefore, in this study, we selected bifidobacterial strains that exhibited high potential as probiotics, had high cell viability in simulated digestive tract conditions, and had good adherence ability with the intestinal epithelial cells. In addition, we selected a strain that could substantially induce IgA secretion in Peyer’s patch cells and promote the expression of tight junction-related genes in the intestinal epithelial cells. Eventually, the effect of this strain on intestinal integrity and microbiota restoration was measured in a mouse model with a kanamycin-induced disturbed intestinal microbial environment.

## Materials and Methods

### Preparation of the Bifidobacterial Strains

*Bifidobacterium* (*B*) *breve* HY3016, *B. breve* HY8921, *Bifidobacterium longum* HY3090, *B. longum* HY3181, *B. longum* HY8805, *Bifidobacterium animalis* ssp. *lactis* HY8002, *B. animalis* ssp. *lactis* HY8901, and *Bifidobacterium infantis* HY8941 were isolated from the feces of only breastfed infants and stored in a seed cell library at Hy Co., Ltd. (Yongin, South Korea). The well-known probiotic *B. animalis* ssp. *lactis* BB12 (ATCC 27536) was used as a reference strain for our *in vitro* experiments (Jungersen et al., 2014). All strains were anaerobically cultured in a blood glucose liver (BL) medium (KisanBio, Seoul, South Korea) at 37°C for 18 h and passaged twice before the experiment. Thereafter, the cultured cells were centrifuged at 4,000 × *g* at 4°C for 5 min and washed twice with saline. Subsequently, the cell pellets were resuspended in saline or phosphate-buffered saline (PBS) for *in vitro* and *in vivo* experiments.

### Evaluation of Strain Viability in Simulated Gastrointestinal Tract Conditions

The probiotic potential of bifidobacterial strains were evaluated by measuring their survival rate in physiological conditions similar to those of the human gastrointestinal tract (GIT). This experiment was performed as previously reported (Kim et al., 2021). Briefly, 5 ml of the culture suspensions (1.0 × 10^9^ CFU/ml in saline) was poured into 50 ml conical tubes. Following this, 26 μl of 0.3 M CaCl_2_ solution and 4 ml of 6.55 mg/ml α-amylase solution were added to each suspension. The physiological conditions of the oral cavity were simulated by adding 1 M NaOH to adjust the pH of the suspensions to 7.0, followed by incubation at 37°C for 2 min. Subsequently, the conditions of the gastric tract were simulated by adding 6 μl of 0.3 mol/L CaCl_2_, 694 μl water, and 9.1 ml of 0.07 mg/ml pepsin to the above mixture. The pH was adjusted to 3 by adding 1 M HCl to the mixture and incubating it at 37°C with continuous shaking for 2 h. Eventually, the intestinal conditions were simulated by adding 40 μl of 0.3 M CaCl_2_, 1.31 ml of distilled water, 2.5 ml of 160 mM bile extract, and 16 ml of 22 mg/ml pancreatic solution to the mixture. The pH of the mixture was adjusted to 7.0 using 1 M NaOH and incubated at 37°C for 2 h. Aliquots of the mixture were collected at the end of each digestive step and subsequently determined the cell viabilities using BL agar plates (Difco, Sparks, MD, United States).

### Determination of Bifidobacterial Adhesion to Intestinal Epithelial Cells

The ability of *Bifidobacterium* strains to adhere to the human intestinal epithelial cells was evaluated by slightly modifying previously reported methods (Shang et al., 2020; Kim et al., 2021). For this purpose, the human colorectal adenocarcinoma cell line Caco-2 was purchased from the American Type Culture Collection (Manassas VA, United States) and was cultured in a Modified Eagle Medium (MEM; Thermo Fisher, Waltham, MA, United States) supplemented with 10% heat-inactivated fetal bovine serum (FBS) at 37°C and 5% carbon dioxide (CO_2_). Subsequently, 1.0 × 10^5^ Caco-2 cells/well were inoculated in a 24-well plate, and the medium was replaced with FBS-free MEM once the cells grew to a 100% confluency. The *Bifidobacterium* strains (test and reference strains) were diluted in PBS, inoculated at 1.0 × 10^8^ CFU/ml of Caco-2 cells/well, and incubated in 5% CO_2_ at 37°C for 2 h. Post-incubation, the cells were washed four times with PBS and separated from the plate by treatment with 0.05% trypsin–EDTA (Sigma-Aldrich, St. Louis, MO, United States) for 5 min. The Caco-2 cell count was obtained using an automated cell counter (Bio-Rad Laboratories, Hercules, CA, United States) and the cell viability of the *Bifidobacterium* spp. was estimated using BL agar plates.

### Animals

Six-week-old specific pathogen-free male BALB/c mice were purchased from DooYeol Biotech (Seoul, South Korea) and maintained in a testing facility at Hy Co., Ltd. for 1 week before experiments were initiated. Animals were housed under controlled conditions: 23 ± 2°C temperature, 50 ± 5% humidity, and a 12-h light/dark cycle (7 am to 7 pm). The mice were fed with sterile AIN-93G (Dyets, Bethlehem, Palestine), and a UV sterilizer and an autoclave were used to sterilize the breeding materials before use. No abnormalities that could possibly affect the experimental outcomes were found in the mice.

#### Ethics Approval Statement

The animal study was reviewed and approved by the Institutional Animal Care and Use Committee of hy Co., Ltd. (approval number: AEC-2021-00008-Y).

### Isolation of Peyer’s Patch Cells and Analysis of IgA Production

The BALB/c mice were sacrificed by CO_2_ overdose and their small intestines were removed. Thereafter, Peyer’s patches were separated using 4 μm micro-scissors. These patches were transferred to an RPMI 1640 medium (Thermo Fisher) supplemented with 1% antibiotic–antimycotic (Thermo Fisher) and were subsequently disrupted by a sterile 100 μm mesh to isolate Peyer’s patch cells. The dissociated cells were aspirated, washed twice with Hank’s balanced salt solution (Sigma-Aldrich), suspended in 10% RPMI 1640 medium, and eventually inoculated in a 96-well plate at a concentration of 1.0 × 10^6^ cells/well. Thereafter, 1.0 × 10^8^ CFU/ml/well of the *Bifidobacterium* strains were incubated at 37°C for 48 h to induce IgA secretion by Peyer’s patch cells. Lipopolysaccharide (LPS) was used as a positive control for the induction of IgA secretion (Kim et al., 2016; Park et al., 2020). Subsequently, the plates were centrifuged and the levels of IgA secreted into the medium were estimated using a mouse IgA ELISA kit (Abcam, Cambridge, United Kingdom).

### Analysis of Tight Junction-Related Gene Expression in CaCo-2 Cells

The expression of the tight junction-related genes, such as *ZO-1* and *OCLN*, was determined by quantitative PCR (qPCR), as follows. Caco-2 cells were cultured to 100% confluency in six-well plates. Next, *Bifidobacterium* strains were resuspended in an antibiotic-free MEM supplemented with 1 μg/ml LPS and incubated with Caco-2 cells at 37°C in a humidified incubator with 5% CO_2_ for 24 h. The Caco-2 cells cultured in MEM without microbial inoculation were designated as the control group. Post-incubation, total RNA was extracted from the CaCo-2 cells using the TRIzol reagent (Sigma-Aldrich) according to the manufacturer’s instructions. Subsequently, cDNA was synthesized using a reverse transcription kit (Qiagen, Hilden, Germany). The mRNA levels were measured using TaqMan Universal PCR Master Mix (Applied Biosystems, Foster, CA, United States) and the QuantStudio 6 Real-Time PCR System (Applied Biosystems). The TaqMan probes used to measure the mRNA expression levels are presented in Table 1. The mRNA expression of glyceraldehyde-3-phosphate dehydrogenase (GAPDH) was used to normalize the expression levels of each target gene (Kozera and Rapacz, 2013).

**Table 1:** TaqMan probes for human genes.

Gene name	TaqMan® probe ID	Dye	Manufacturer
Human GAPDH (*GAPDH*)	Hs02786624_g1	FAM	Thermo Fisher Scientific
Human Zonula occludens 1 (*ZO-1*)	Hs01551861_m1	FAM	
Human occludin (*OCLN*)	Hs05465837_g1	VIC	

### Design and Treatment for Animal Experiments

**Figure 1:**
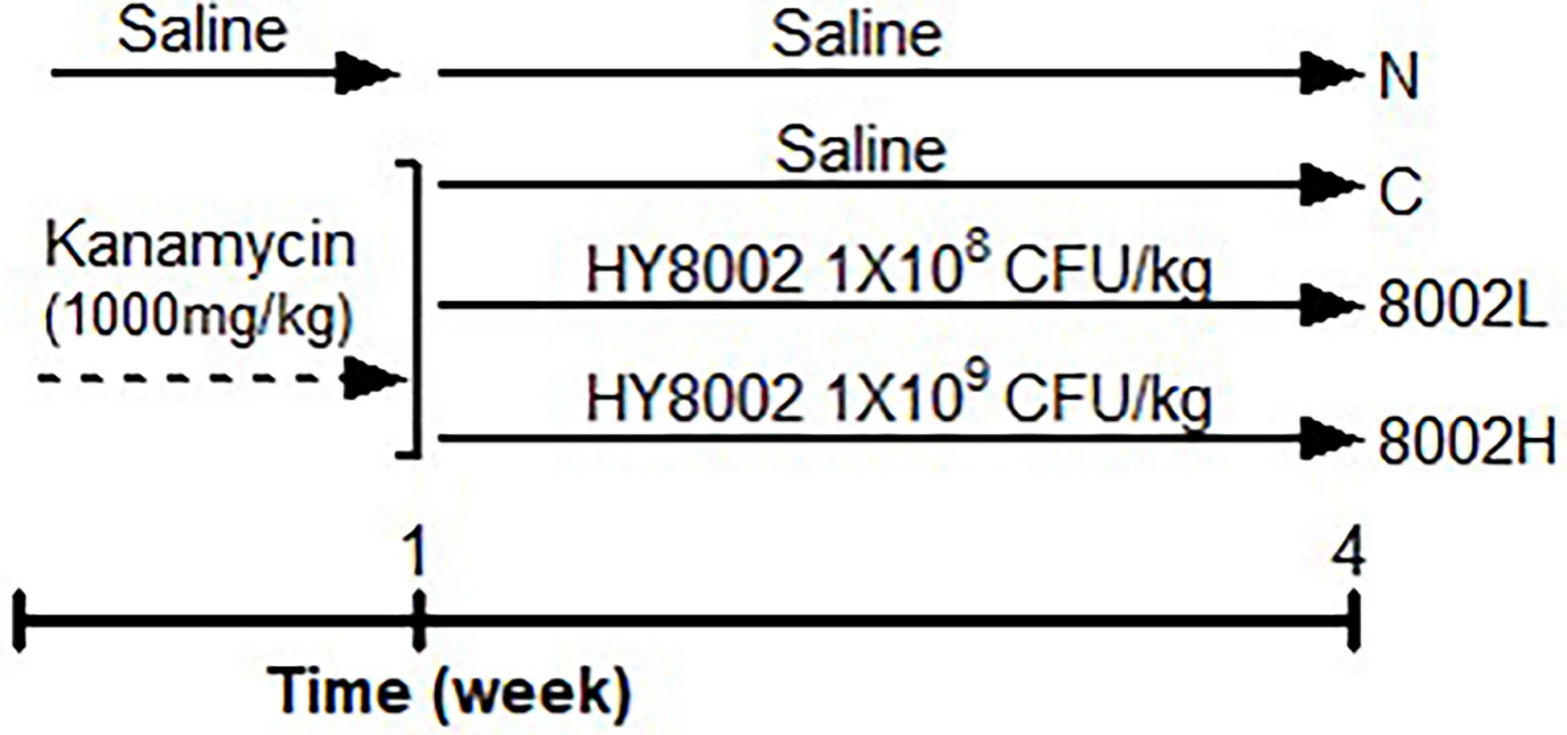
Timeline and treatments of the animal experiments. N, normal group; C, kanamycin administration group; 8002L, kanamycin with 1.0 × 10^8^ CFU/kg/day HY8002 administration group; 8002H, kanamycin with 1.0 × 10^9^ CFU/kg/day HY8002 administration group.

Seven-week-old male BALB/c mice were divided into four groups (*n* = 8 per group): normal group (N), 1,000 mg/kg/day kanamycin administration group (C), kanamycin with 1.0 × 10^8^ CFU/kg/day HY8002 administration group (8002L), and kanamycin with 1.0 × 10^9^ CFU/kg/day HY8002 administration group (8002H). To induce intestinal dysbiosis, kanamycin was orally administered to all mice except the N group for 7 consecutive days. On the other hand, mice in the normal group were administered with only physiological saline (0.9% NaC). After kanamycin treatment, HY8002 suspended in physiological saline was orally administered to the HY8002L and HY8002H groups for 4 weeks, and only physiological saline was administered to the N and C groups during the same period (Figure 1). No animals died or exhibited any abnormal characteristics during the experiment. Food intake and body weight of the mice were measured once per week until the mice were sacrificed.

### Total Cell Count of Peyer’s Patches and Measurement of Intestinal IgA Levels

Post-treatment, mice were euthanized by CO_2_ overdose and their small intestines and cecum were dissected. Peyer’s patch cells were isolated from the dissected small intestine using the method specified in Animals section. Subsequently, the total cell count was determined using an automatic cell counter (Bio-Rad). Upon isolating Peyer’s patches, the remaining small intestines were stored at −80°C for mRNA analysis. Additionally, feces from the isolated cecum were collected, and the secreted IgA levels were measured using a mouse IgA ELISA kit (Abcam).

### Measurement of Plasma Immunoglobulins and Cytokines

Before the mice were sacrificed, their blood was isolated from the abdominal vein using a syringe and isolated the plasma using EDTA tubes (BD, Franklin Lakes, NJ, United States). Thereafter, the blood immunoglobulin (such as IgA, IgG2a, and IgE) concentration in the isolated plasma (Kim et al., 2016; Park et al., 2020) was determined using an ELISA kit (Abcam) according to the manufacturer’s instructions. The levels of pro-inflammatory cytokines tumor necrosis factor alpha (TNF-α), interleukin 1beta (IL-1β) and interferon gamma (IFN-γ), and anti-inflammatory cytokine Interleukin 10 (IL-10) in plasma were measured using the Multiplex Cytokine Assay Kit (Mouse Luminex Discovery Assay, R&D systems, Minneapolis, MN, United States).

### Measurement of mRNA Levels in the Intestinal Tissue

The mRNA levels of ZO-1, OCLN, TLR2, and TLR4 were measured by qPCR. Briefly, total RNA was extracted from the small intestine tissues using the AllPrep RNA mini kit (QIAGEN Sciences Inc., Germantown, MD, United States) as per the manufacturer’s instructions. Subsequently, cDNA synthesis and qPCR were performed as described in Analysis of Tight Junction-Related Gene Expression in CaCo-2 Cells section. The TaqMan probes used to measure mRNA levels in the tissues of the small intestine are presented in Table 2.

**Table 2:** TaqMan probes for mouse genes.

Gene name	TaqMan® probe ID	Dye	Manufacturer
Mouse GAPDH (*GAPDH*)	Mm99999915_g1	FAM	Thermo Fisher Scientific
Mouse Zonula occludens-1 (*ZO-1*)	Mm01320638_m1	FAM	
Mouse occludin (*OCLN*)	Mm00500912_m1	FAM	
Mouse Toll-like receptor 2 (*TLR2*)	Mm01213946_g1	FAM	
Mouse Toll-like receptor 4 (*TLR4*)	Mm00445273_m1	FAM	

### Fecal Microbiota Analysis

The feces (0.3 g per mouse) of four different mice were randomly collected from each group prior to sacrificing the animals for gut flora analysis. Total DNA was isolated from the feces, and the microbiota composition was verified by 16S rDNA sequencing using a next-generation sequencing platform (Illumina, San Diego, CA, United States). The universal primer pairs used for the sequencing were as follows: V3-F: 5′-TCGTCGGCAGCGTC AGATGTGTATAAGAGAC AGCCTACGGGNG GCWGCAG-3′ and V4-R: 5′-GTCTCGTGGGCTC GGAGATGTGTATAAGA GACAGGACTACHV GGGTATCTAATCC-3′. Next, DNA extraction, 16S rDNA sequencing, and bioinformatics analysis were performed at Macrogen (Seoul, South Korea). The operational taxonomic units were aligned with the NCBI 16S microbial database, and all the taxonomic information was compiled using the BLAST+ database (v. 2.9.0; Lee et al., 2021). The Chao1 and PD_whole tree indices were analyzed using the QIIME program (Magoc and Salzberg, 2011). Any significant differences in genus levels between groups were analyzed by conducting Linear discriminant analysis Effect Size online.^1^ Correlation analyses were conducted and visualized using the FactoMineR package (available online: https://www.r-project.org accessed on September 10, 2021). Spearman’s correlation analysis was performed to analyze the results statistically by using FactoMineR (Lee et al., 2021).

### Safety Evaluation by Whole-Genome Sequencing

Gelatinase, hyaluronidase, aggregation substance, enterococcal surface protein, cytolysin, enterotoxin, non-hemolytic enterotoxin, hemolysin, cereulide, serine protease, and transposon-related enzymes are well-established as potential virulence factors of microorganisms (Di Cagno et al., 2013; Kim et al., 2021). Analysis of whether HY8002 expressed the genes pertaining to these enzymes was done by performing whole-genome sequencing and bioinformatics analyses in Chunlab (Chunlab Inc., Seoul, South Korea). All datasets have been deposited in NCBI GeneBank with the accession number PRJNA819830.

### Statistical Analyses

Statistical results for our *in vitro* and *in vivo* experiments are expressed as mean ± SEM at the 95% confidence limit. Data were statistically compared by one-way ANOVA and a *post-hoc* Tukey test. All statistical analyses were performed using GraphPad Prism v5 (San Diego, CA, United States).

## Results

### Survival Rate of Bifidobacterial Strains Under Simulated Gastrointestinal Tract Conditions

**Figure 2:**
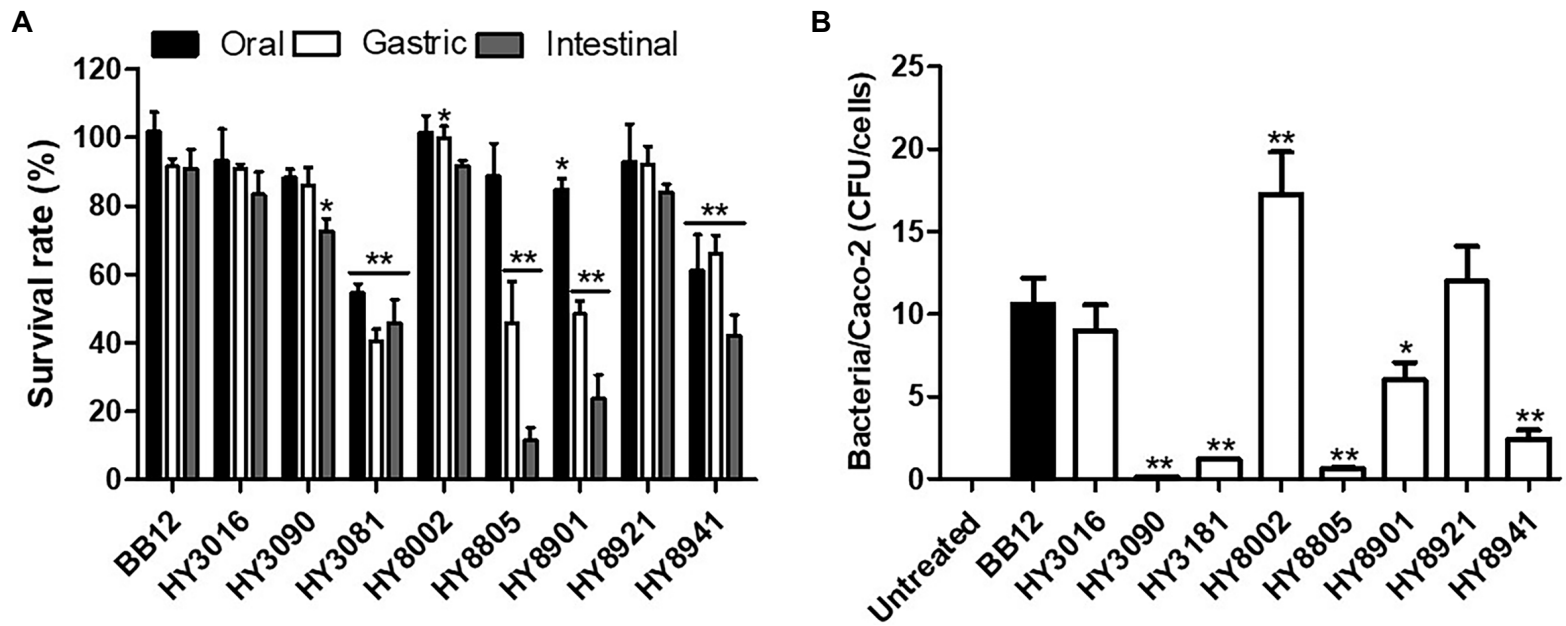
**(A)** Survival rate of bifidobacterial strains in simulated gastrointestinal tract conditions. **(B)** The number of bacterial cells attached to one Caco-2 cell under co-culture conditions. “Bacteria/Caco-2 cells” indicates bacterial colony-forming units (CFU) per Caco-2 cell. Data are represented as mean ± SEM of three independent experiments. ^*^p < 0.05 and ^**^p < 0.01 as compared with BB12.

Upon measuring the survival rate of the *Bifidobacterium* strains under simulated GIT conditions, the artificial saliva treatment was observed to have less effect on bacterial survival compared with the other two digestive juices. In fact, HY8002 exhibited a significantly higher gastric survival rate than the reference strain BB12, whereas HY3016 and HY8921 had survival rates that were similar to that of the reference strain. In contrast, HY3090 and HY8805 strains displayed relatively low resistance to intestinal conditions. Moreover, HY3181, HY8901, and HY8941 had a lower survival rate than BB12 under the simulated GIT conditions (Figure 2A).

### Ability of Bifidobacterial Strains to Adhere to Caco-2 Cells

The *in vitro* test performed to estimate the ability of the bifidobacterial strains to adhere to the intestinal cells revealed that approximately 18 HY8002 cells adhered to the Caco-2 cells for 2 h. On the contrary, approximately only 10 BB12 adhered to the Caco-2 cells. Furthermore, HY3090, HY3181, HY8805, and HY8941 exhibited very low rates of adhesion to CaCo-2 cells, whereas HY3016 and HY8921 had adhesion rates similar to that of BB12. Therefore, HY8002 was validated to display the highest ability to adhere to intestinal epithelial cells among all the tested strains, including the reference strain (Figure 2B).

### Effects of Bifidobacterial Strains on Immunoglobulin A Production *in vitro*

**Figure 3:**
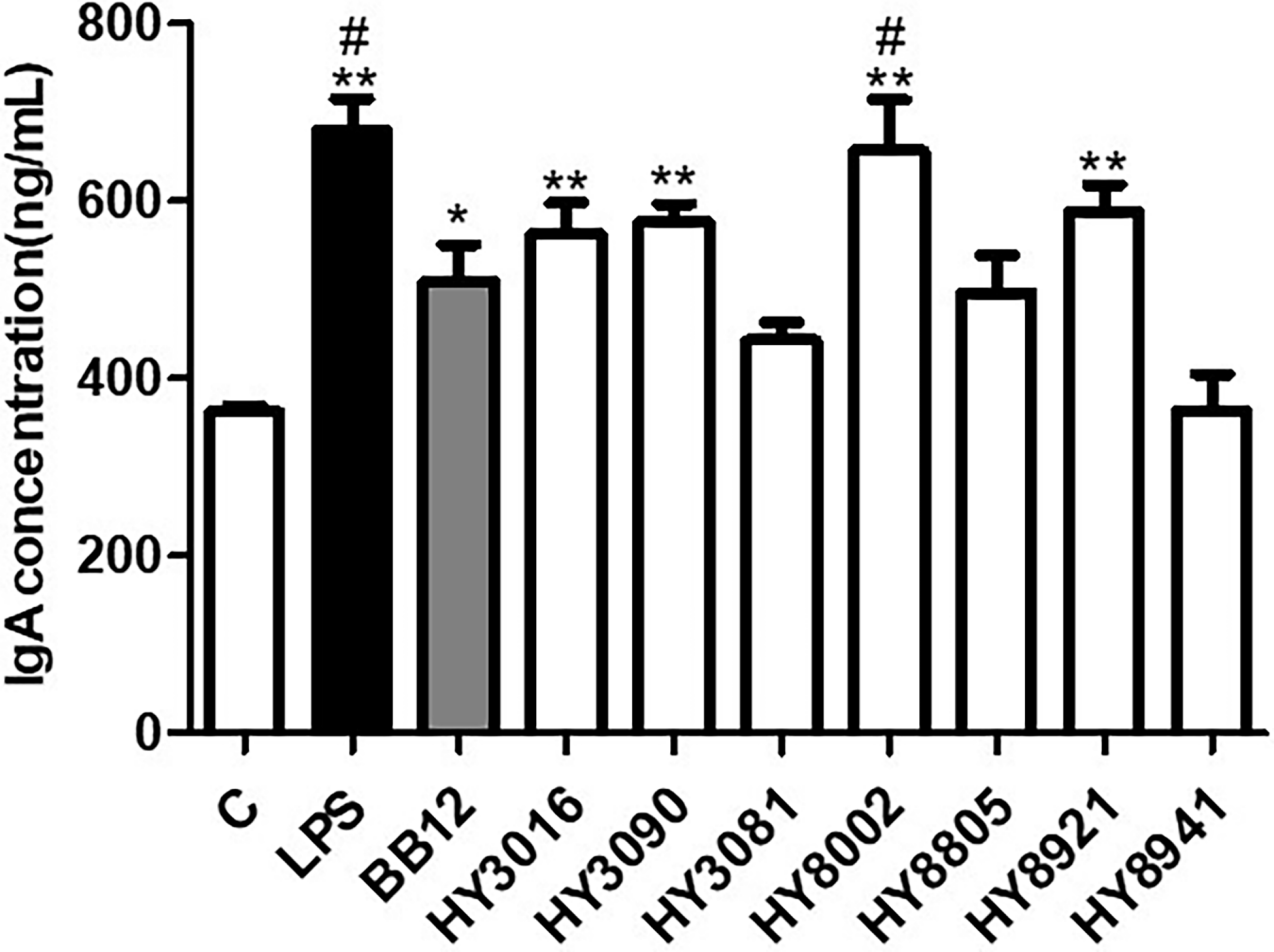
Effect of *Bifidobacterium* strains on immunoglobulin A (IgA) production by Peyer’s patch cells. Lipopolysaccharide was used as the positive control for induction of IgA secretion. The IgA concentration in the culture media was determined by ELISA. Each value is expressed as the means ± SEM of three independent experiments. ^*^p < 0.05 and ^**^p < 0.01 in comparison with the untreated control. ^#^*p* < 0.05 compared with BB12 treated.

To predict the effect of *Bifidobacterium* strains on intestinal immune regulation, the levels of IgA produced by Peyer’s patch cells were measured. Barring HY3081, HY8805, and HY8941, most strains were found to enhance IgA secretion in these cells. Notably, HY8002 was observed to significantly induce higher IgA secretion than the reference strain. Nonetheless, no strains induced higher IgA secretion than the positive control LPS (Figure 3).

### Effects of Bifidobacterial Strains on Tight Junction-Related Gene Expression in Caco-2 Cells

**Figure 4:**
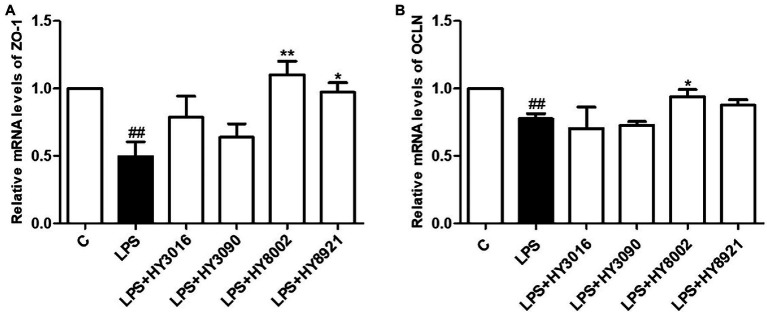
Effect of *Bifidobacterium* strains on **(A)** zonula occludens-1 mRNA expression **(B)** and occludin mRNA expression in lipopolysaccharide (LPS)-treated Caco-2 cells. Each value is expressed as the mean ± SEM of three independent experiments. ^*^p < 0.05 and ^**^p < 0.01 compared with LPS-treated. ^##^*p* < 0.01 compared with untreated control.

The strains HY3016, HY3090, HY8002, and HY8921 were selected to determine the effect of bifidobacterial strains on tight junction-related gene expression in intestinal epithelial cells. This was because these strains remarkably induced IgA secretion and had substantial cell viability under GIT conditions. Moreover, they exhibited superior or similar magnitude of adherence to Caco-2 cells than the reference strain. While LPS treatment significantly reduced the mRNA levels of ZO-1 and OCLN in Caco-2 cells, HY8002 and HY8921 considerably restored the mRNA levels of ZO-1 (Figure 4A). However, the mRNA levels of OCLN were increased only in the HY8002 treated groups (Figure 4B).

### Effect of HY8002 on the Food Intake, Body Weight, and Spleen Indices of Mice

**Figure 5:**
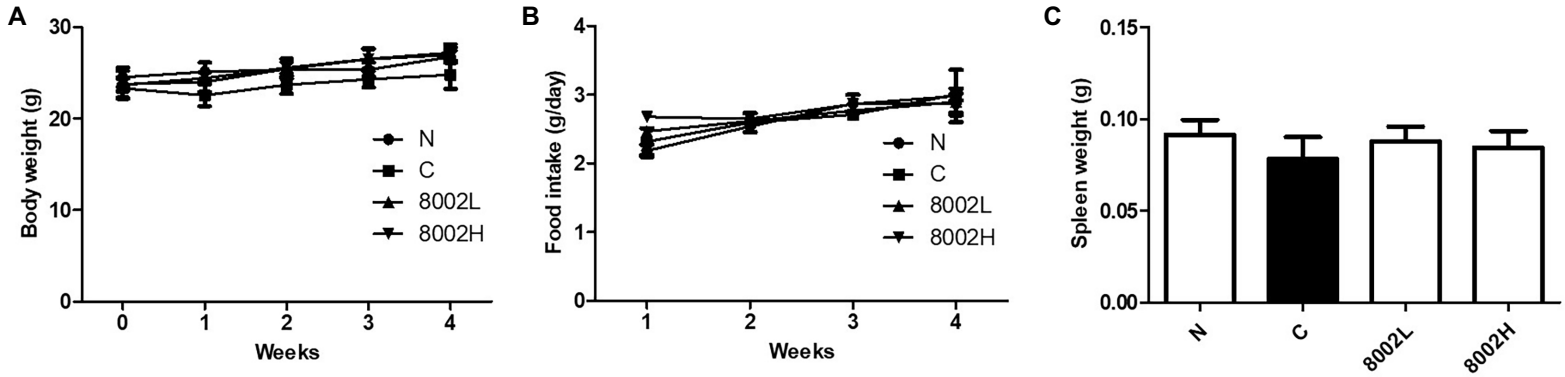
Effect of HY8002 administration on **(A)** body weight, **(B)** food intake, and **(C)** spleen tissue mass in the kanamycin-treated mice. The results are expressed as the mean ± SEM. N, normal group (*n* = 8); C, kanamycin administration group (*n* = 8); 8002L, kanamycin with 1.0 × 10^8^ CFU/kg/day HY8002 administration group (*n* = 8); 8002H, kanamycin with 1.0 × 10^9^ CFU/kg/day HY8002 administration group (*n* = 8).

Since HY8002 had the highest cell viability under GIT conditions and the most adherence to Caco-2 cells and induced the highest IgA secretion among all the bifidobacterial strains, a follow-up experiment was conducted to investigate its effect on intestinal integrity. For this purpose, a kanamycin-treated mouse model was used, and high kanamycin concentration was observed to slightly reduce the mouse body weight (Figure 5A). However, HY8002 administration restored this weight loss; nonetheless, there was no significant difference between all the groups. Moreover, no significant difference in food intake was observed between the normal group, the kanamycin-treated control group, and the HY8002 administered groups (Figure 5B). Although the weight of the mouse spleen decreased in the kanamycin-treated group and increased in the HY8002 treated groups, this difference was statistically insignificant (Figure 5C).

### Effects of HY8002 on the Composition of Peyer’s Patches in the Small Intestine

**Figure 6:**
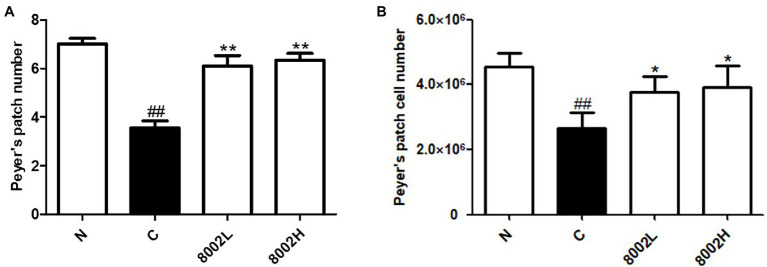
Effect of HY8002 administration on the composition of Peyer’s patch structures, including **(A)** number of Peyer’s patches and **(B)** total number of Peyer’s patch cells in kanamycin-treated mice. The results are expressed as the mean ± SEM. ^*^p < 0.05 and ^**^p < 0.01 compared with kanamycin-treated control groups. ^##^*p* < 0.01 compared with normal groups. N, normal group (*n* = 8); C, kanamycin administration group (*n* = 8); 8002L, kanamycin with 1.0 × 10^8^ CFU/kg/day HY8002 administration group (*n* = 8); 8002H, kanamycin with 1.0 × 10^9^ CFU/kg/day HY8002 administration group (*n* = 8).

As shown in Figure 6A, the number of Peyer’s patches in kanamycin-treated mice was significantly lower than that in normal mice. On the other hand, HY8002 administration was verified to increase the number of Peyer’s patches to a value similar to that observed in the normal group. The same trend was observed for the total number of Peyer’s patch cells (Figure 6B). However, no difference in the number of Peyer’s patches and the total number of Peyer’s patch cells between the 8002L and the 8002H groups was observed.

### Effects of HY8002 on *in vivo* Immunoglobulin and Cytokine Production

**Figure 7:**
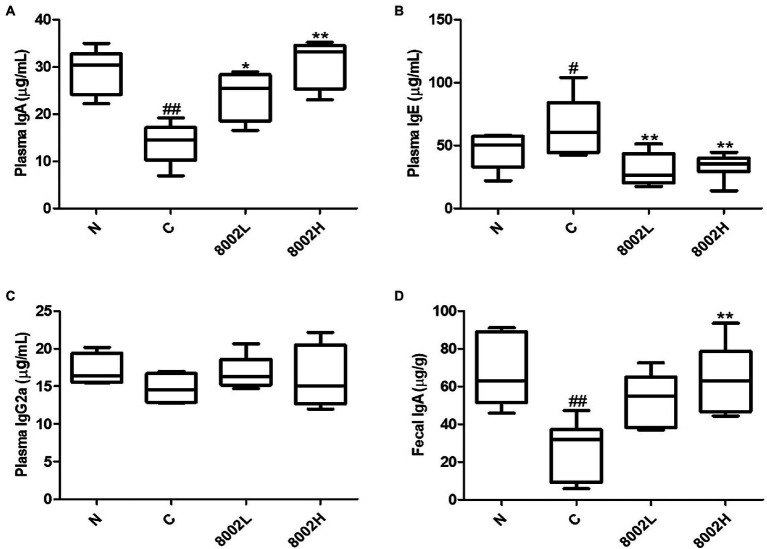
Effect of HY8002 administration on plasma **(A)** IgA, **(B)** IgE, **(C)** IgG2a, and **(D)** fecal IgA in kanamycin-treated mice. The results are expressed as Box and Whisker Plots (median with minimum to maximum values). ^*^p < 0.05 and ^**^p < 0.01 compared with kanamycin-treated control groups. ^#^*p* < 0.05 and ^##^*p* < 0.01 compared with normal groups. N, normal group (*n* = 8); C, kanamycin administration group (*n* = 8); 8002L, kanamycin with 1.0 × 10^8^ CFU/kg/day HY8002 administration group (*n* = 8); 8002H, kanamycin with 1.0 × 10^9^ CFU/kg/day HY8002 administration group (*n* = 8).

Studies have reported that high-dose antibiotics treatment weakens the intestinal mucosal barrier by decreasing intestinal IgA levels. It also reduces plasma IgA and IgG2a levels and increases IgE levels, causing systemic immune dysfunction (Sudo et al., 2002; Kim et al., 2016). While kanamycin significantly decreased the plasma IgA levels, HY8002 administration recovered these levels to those in the untreated mice (Figure 7A). The same trend was observed for fecal IgA levels (Figure 7D). These effects exhibited a HY8002 dose-dependent trend. Additionally, the plasma IgE levels in the control group were significantly higher than those in the normal group, even 3 weeks post the final kanamycin administration. However, this elevation was significantly suppressed in the HY8002 groups (Figure 7B). On the other hand, there were no significant differences in IgG2a levels among all the treatment groups (Figure 7C). Cytokines may positively or negatively affect intestinal epithelial barrier integrity and may originate from innate or adaptive immune cells or from the intestinal epithelial cells (Andrews et al., 2018). Blockade of the inflammatory cytokines TNF-α and IL-1β has been studied as a target for the treatment of inflammatory bowel disease (IBD) and ulcerative colitis (UC; Pizarro et al., 2021). IL-10 is an anti-inflammatory cytokine known to have a positive role in maintaining intestinal immune homeostasis, and it is known that intestinal inflammation occurs spontaneously in mice lacking the *IL-10* gene (Alfen et al., 2018). IFN-γ is an immunoregulatory cytokine involved in regulating macrophage activation and T helper cell differentiation. Levels of IFNγ are often elevated locally and systemically in chronic inflammatory diseases, including IBD, resulting in disruption of intestinal barrier function accompanied by decreased expression of the tight junction molecules ZO-1 and occluding (Smyth et al., 2011). Concentrations of TNF-α and IL-1β in plasma were measured at similar levels of all groups including the kanamycin-treated group (Supplementary Figures 1A,B). IFN-γ increased in the kanamycin group and tended to decreased in the HY8002 administration group (Supplementary Figure 1C). In contrast, plasma IL-10 levels tended to decrease in the kanamycin administration group and to increase in the HY8002 administration group (Supplementary Figure 1D). However, there was no statistical significance in IFN-γ and IL-10 levels between all treatment groups.

### Effects of HY8002 on mRNA Levels of Tight Junction-Related Genes and Toll-Like Receptor Genes in the Small Intestine

**Figure 8:**
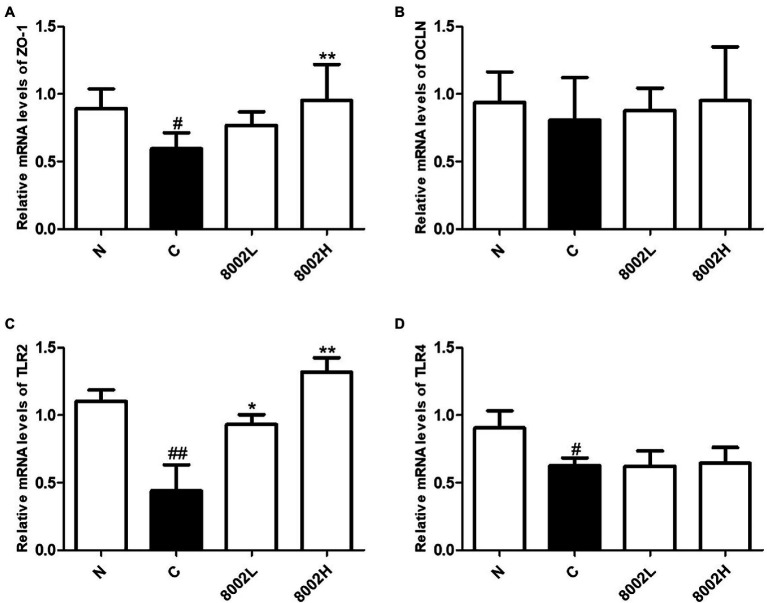
The mRNA levels of **(A)** ZO-1, **(B)** occludin, **(C)** TLR2, and **(D)** TLR4 in small intestine. Data are represented as mean ± SEM. ^*^p < 0.05 and ^**^p < 0.01 compared with kanamycin-treated control groups. ^#^*p* < 0.01 and ^##^*p* < 0.01 compared with normal groups. N, normal group (*n* = 8); C, kanamycin administration group (*n* = 8); 8002L, kanamycin with 1.0 × 10^8^ CFU/kg/day HY8002 administration group (*n* = 8); 8002H, kanamycin with 1.0 × 10^9^ CFU/kg/day HY8002 administration group (*n* = 8).

The mRNA levels of the tight junction gene *ZO-1* and the genes encoding the pattern recognition receptors TLR2 and TLR4 were significantly decreased by kanamycin treatment in the mouse small intestine (Figures 8A–D). However, the mRNA levels of OCLN were not significantly reduced (Figure 8B). Notably, the mRNA level of ZO-1 was significantly upregulated in the 8002H group and the mRNA level of TLR2 was significantly upregulated in both the 8002L and 8002H groups (Figures 8A,C). In contrast, the mRNA levels of TLR4 were unaffected by HY8002 administration (Figure 8D).

### Effect of HY8002 on Intestinal Microbial Diversity and Microbial Profiles in Kanamycin-Treated Mice

**Figure 9:**
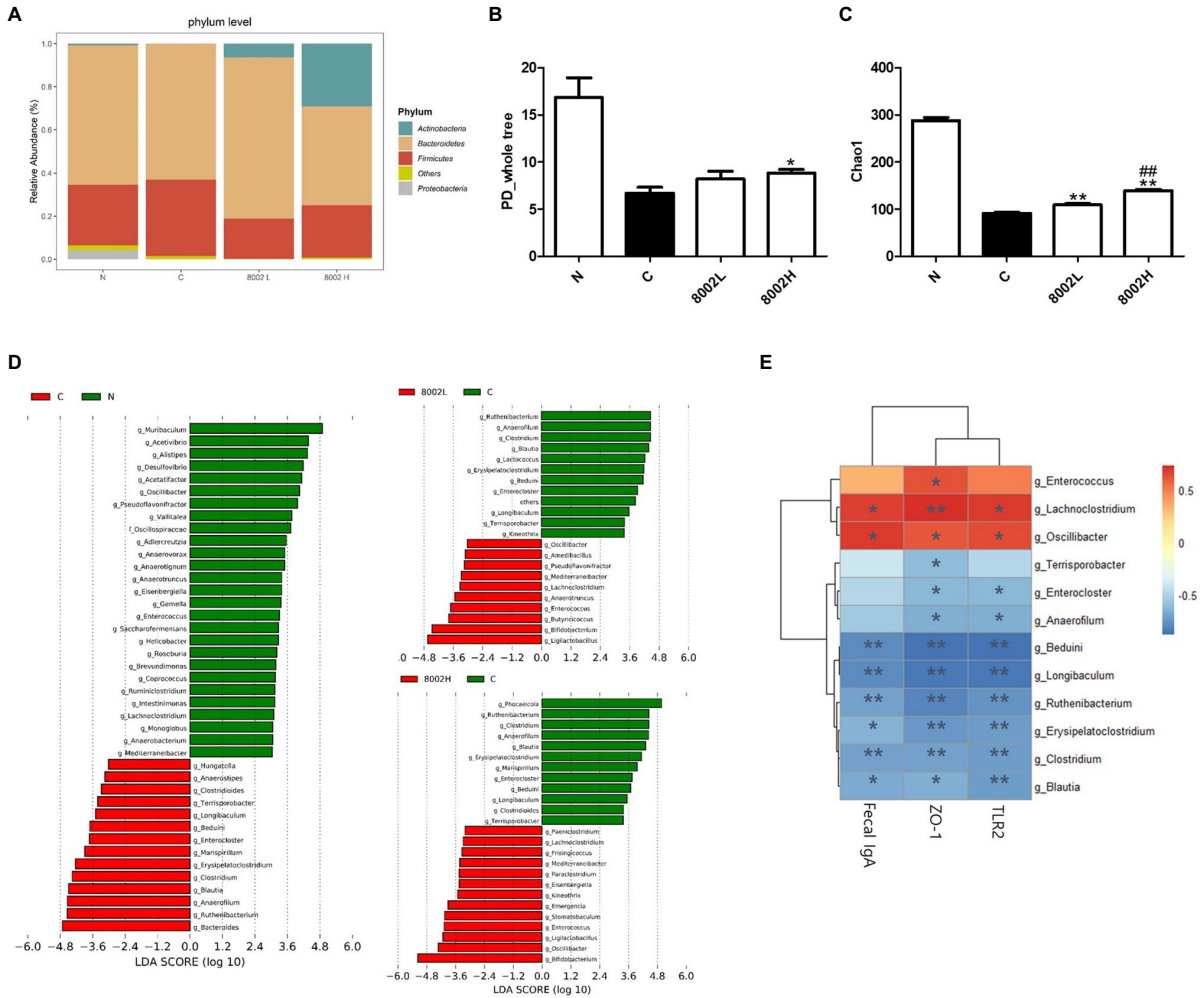
Effect of HY8002 administration on the composition of bacterial taxa in mouse feces. **(A)** Relative abundance of phylum level in each treatment group. The α-diversity of each group: **(B)** PD_whole tree index and **(C)** Chao1 index. **(D)** Histogram of the linear discriminant analysis (LDA) scores for differentially abundant taxa following kanamycin treatment. **(E)** Correlation between bacterial genus-level taxa and measured intestinal integrity-related factors. Statistical analysis was conducted by the spearman correlation analysis. Data are represented as mean ± SEM. ^*^p < 0.05 and ^**^p < 0.01. ^##^*p* < 0.01 compared with 8002L groups. N, normal group (*n* = 4); C, kanamycin administration group (*n* = 4); 8002L, kanamycin with 1.0 × 10^8^ CFU/kg/day HY8002 administration group (*n* = 4); 8002H, kanamycin with 1.0 × 10^9^ CFU/kg/day HY8002 administration group (*n* = 4).

The effect of HY8002 on the intestinal environment was investigated by analyzing the differences in the intestinal microbial composition in each treatment group and comparing these differences within a specific microbial taxon. As shown in Figure 9A, Firmicutes comprised 28.1% and 35.5% of the total intestinal microbiome in the normal and control groups, respectively. Additionally, they amounted to 18.7% and 24.2% in the 8002L and 8002H groups, respectively. In contrast, kanamycin did not alter the percentage of Bacteroidetes in the microbiome (64% in the normal group and 63% in the control group). Proteobacteria were lower in number in the control, 8002L, and 8002H groups than in the normal group. Actinobacteria (the phylum that includes the *Bifidobacterium* spp.) were significantly decreased by kanamycin treatment; however, their numbers increased in a dose-dependent manner following HY8002 treatment. The α-diversity index, an indicator of microbial diversity within a group, was substantially lowered upon kanamycin treatment but was significantly improved upon HY8002 administration (Figures 9B,C). Notably, the Cho1 index was higher in the 8002H group than that in the 8002L group (Figure 9C). The linear discriminant analysis (LDA) scores for differentially enriched taxa post-kanamycin and HY8002 treatments are presented in Figure 9D. While the relative abundance of the *Oscillibacter* genus was significantly decreased in the control group, high-dose HY8002 administration significantly restored this abundance (Supplementary Figure 2A). In contrast, the relative abundances of the *Clostridium* and *Blautia* genera were significantly higher in the control group than that in the normal group. Moreover, HY8002 administration (at all doses) restored these levels to those in the normal group (Supplementary Figures 2B,C). Eventually, a Spearman correlation analysis between the mucosal integrity-related indicators of the intestine (e.g., intestinal immunity and tight junctions) and the genus-level taxa that contributed the most to the LDA (except for *Bifidobacterium* spp.; Figure 9E) was performed. The *Lachnoclostridium* and *Oscillibacter* genera were observed to be positively correlated with the ZO-1 and TLR2 mRNA levels and IgA secretion in the small intestine. On the contrary, the *Beduini*, *Longibaculum*, *Ruthenibacterium*, *Erysipelatoclostridium*, *Clostridium*, and *Blautia* genera were negatively correlated with the ZO-1 and TLR2 mRNA levels and IgA secretion in the small intestine. The *Enterococcus* and *Terrisporobacter* genera had positive and negative correlations with ZO-1 expression, respectively.

### Effects of Toll-Like Receptors on HY8002-Induced IgA Secretion and *ZO-1* Expression

**Figure 10:**
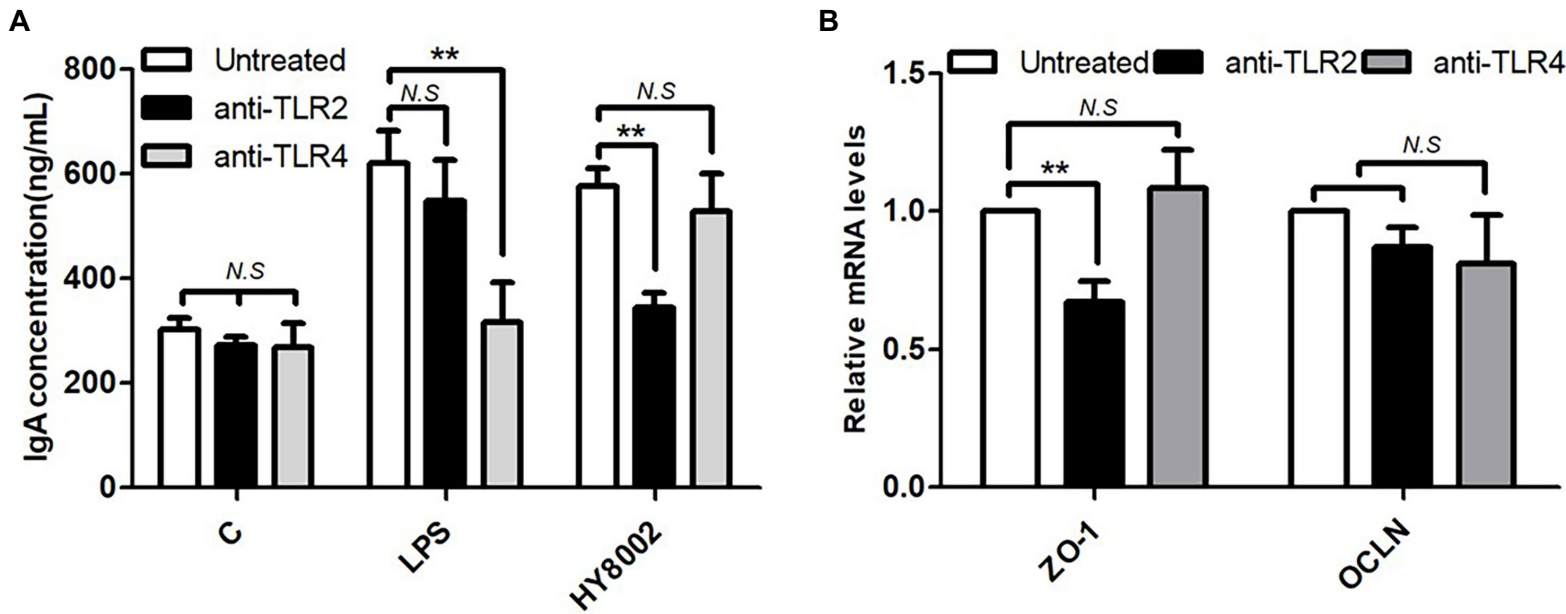
Effect of TLR2 and TLR4 blockade by antibodies on **(A)** IgA production levels in Peyer’s patch cells stimulated with HY8002 and **(B)** the mRNA levels of tight junction molecules in HY8002 treated Caco-2 cells. Data are represented as mean ± SEM. ^**^p < 0.01 compared with antibody untreated groups.

In the above *in vivo* experiment, it was confirmed that administration of HY8002 restored the levels of TLRs, especially TLR2, which were reduced by kanamycin treatment. It was also found that the intestinal microbiota affected by kanamycin and/or HY8002 correlated with IgA secretion and ZO-1 and TLR2 levels in small intestine. Therefore, whether HY8002 directly affects IgA secretion and ZO-1 levels through TLR signaling was investigated using blocking antibodies. After treatment with 0.1 μg/ml of TLR2 and TLR4 blocking antibodies (InvivoGen, San Diego, CA, United States) to Peyer’s patch cells isolated from the BALB/c mouse small intestine, the degree of IgA secretion by HY8002 was measured. As a result, IgA secretion was significantly reduced by the TLR2 antibody. In contrast, IgA secretion by LPS was decreased by TLR4 blockade (Figure 10A). As a result of confirming the mRNA change of the tight junction molecules by HY8002 in Caco-2 cells pretreated with TLR2 and TLR4 blocking antibody, ZO-1 was significantly reduced by TLR2 blocking. The mRNA levels of OCLN tended to be slightly decreased by TLR2 and TLR4 blockade, but there was no statistical significance (Figure 10B).

### Biological Safety Assessment of HY8002 Based on Whole-Genome Sequencing

The total length of the HY8002 chromosomal DNA was 1,944,140 bp with 60.5% GC content and 1,563 predicted open reading frames. Additionally, genome annotation identified the major genes to be *beta-galactosidase*, *beta-cylosidase*, and *gamma-glutamylcysteine synthetase* involved in glycoside degradation, carbohydrate metabolism, and glutathione synthesis, respectively (Supplementary Figure 3). A phylogenetic tree was constructed based on the average nucleotide identity value, and HY8002 was confirmed to be similar to bifidobacterial probiotic strains, such as *Bifidobacterium animalis* ssp. *lactis* DSM10140 (Ehrmann et al., 2003), *Bifidobacterium animalis* ssp. *lactis* V9 (Sun et al., 2010), and *Bifidobacterium animalis* ssp. *lactis* B420 (Stahl and Barrangou, 2012; Supplementary Figure 4). In addition, it was established that HY8002 expressed no commonly known virulence genes (data not shown).

## Discussion

Scientific evidence that symbiotic and probiotic bacteria play an important role in maintaining and promoting gut homeostasis and intestinal epithelial barrier function is steadily increasing (Lee et al., 2019; Invernici et al., 2020; Ojima et al., 2020; Al-Sadi et al., 2021). Recently, studies have indicated that the intestinal tight junction barrier and immune response are therapeutic targets for the management of several diseases (Lee et al., 2018; Zeisel et al., 2019; Sun and Zhou, 2020). Of note, among the various probiotic bacteria reported to date, *Bifidobacterium* spp. are among the most widely used probiotic bacteria. They colonize the human intestine approximately first and account for approximately 80% of the gut microbiota of breastfed infants (Al-Sadi et al., 2021). Their health-promoting activities range from modulating the host immune response, strengthening the intestinal barrier, inhibiting pathogenic bacteria, and maintaining gut homeostasis by building a healthy microbiome. Studies have demonstrated *Bifidobacterium* spp. to be innate and essential members of the human gut, necessary for maintaining the integrity of the intestinal epithelial barrier (Lee et al., 2019; Invernici et al., 2020; Ojima et al., 2020; Shang et al., 2020; Al-Sadi et al., 2021). A recent study reported that gut microbiome profiles of patients with ulcerative colitis exhibited a significant reduction in *Bifidobacterium* spp. (Duranti et al., 2016). However, the beneficial effects of the *Bifidobacterium* genus in the treatment of various diseases remains a controversy (Al-Sadi et al., 2021). This is because there is a gap in scientific knowledge regarding which *Bifidobacterium* spp. is responsible for strengthening the intestinal barrier and which spp. or strain has the greatest effect. There is also a report that the effect of Bifidobacterium on immunomodulation differs depending on the strain, not the species (Medina et al., 2007).

In this study, our primary aim was to identify specific *Bifidobacterium* strains that preserve intestinal integrity by enhancing intestinal mucosal immunity and tight junction barrier function and evaluate their potential as probiotics. The secondary goal was to analyze the effects of the selected *Bifidobacterium* strains on the total intestinal microbiota and to analyze the correlation between the microorganisms involved in maintaining intestinal integrity. To this end, we evaluated the digestion resistance and IgA promoting activity of various bifidobacterial strains isolated from the feces of infants. Additionally, we measured the effect of these strains on upregulating the gene expression of *ZO-1* and *OCLN in vitro*. Consequently, we selected HY8002 for subsequent experiments and determined its *in vivo* effect on antibiotics-disrupted intestinal mucosal immunity (IgA production), tight junctions (ZO-1, OCLN, and TLR2), and intestinal microbiome (fecal microbiota by 16 s sequencing analysis).

Although many *Bifidobacterium* strains are used as probiotics, all strains are not probiotics; rather, only strains with gastrointestinal stability and biosafety can be proposed as probiotics (Izquierdo et al., 2008; George Kerry et al., 2018). In fact, the ability to survive in the gastrointestinal tract and attach to the intestinal epithelium is one of the key criteria for probiotic strain selection (Shang et al., 2020; Kim et al., 2021). Orally ingested probiotics reach the intestines through stressful environments, such as saliva, gastric juice, and bile fluids. Therefore, an effective probiotic strain must have high a resistance to digestive conditions and the ability to adhere to the intestine epithelium (Shang et al., 2020). We simulated conditions of the GIT and determined that HY8002 exhibited very high cell viability, particularly in the gastric environment. Moreover, it displayed better digestive stability than the reference BB12 strain (Jungersen et al., 2014) a well-known commercial probiotic strain (Figure 2A). Furthermore, HY8002 adhered well to Caco-2 cells, a human intestinal epithelial cell line (Figure 2B). Additionally, we validated the genetic safety of this strain through whole-genome sequencing (Supplementary Figure 3).

The gastrointestinal tract is the most common entry point for infectious agents, including pathogenic bacteria and viruses, from the mucosal surfaces. The IgA immunoglobulin is a key factor at the forefront of mucosal tissue immunity, including that of the intestine, and serves as a standard for evaluating mucosal immunity (Cunningham-Rundles, 2001; Boullier et al., 2009; Park et al., 2020). Recent studies have confirmed that high-dose broad-spectrum antibiotics reduce IgA secretion because of the death of commensal strains, including intestinal probiotics. This decreases the defense against certain pathogenic bacteria, such as *Pseudomonas aeruginosa* and *Clostridium difficile* (Dzunkova et al., 2016; Robak et al., 2018). Our *in vitro* test determined that HY8002 induced the highest IgA secretion among the five *Bifidobacterium* strains (including BB12) that increased IgA secretion by Peyer’s patch cells (Figure 3). In addition, our animal experiments confirmed that HY8002 restores the number of Peyer’s patches, total cell count of Peyer’s patches, and the IgA levels decreased by kanamycin treatment (Figures 6, 7A,D). Remarkably, HY8002 significantly reduced the IgE levels increased by kanamycin, suggesting that it modulates the antibiotics-induced TH1/TH2 immune imbalance (Figure 7B). Although previous reports have stated that probiotics regulate systemic TH1/TH2 balance (Kim et al., 2016), the overall immunomodulatory effect of HY8002, excluding intestinal immunity, could not be deduced from this study and further studies are needed.

IgA plays a role in the front-line defense of the intestinal mucosa, whereas tight junctions play a role in maintaining a protective barrier in the posterior region in intestinal epithelium (Suzuki, 2013; Gu et al., 2016; Kim et al., 2016; Feng et al., 2019). Tight junction complexes have been predicted to be affected by TLRs, particularly by TLR2 that regulates *ZO-1* gene expression (Cario et al., 2004). Some probiotic strains of the *Lactobacillus* and *Bifidobacterium* genera increase the expression of tight junction-related genes (Kim et al., 2016; Al-Sadi et al., 2021); however, not all probiotic strains improve tight junction-related gene expression (Sultana et al., 2013). In our study, HY8002 significantly increased the mRNA levels of ZO-1 and OCLN reduced by LPS in Caco-2 cells (Figures 4A,B). It also restored the mRNA expression of ZO-1 to normal levels in the small intestine of kanamycin-treated mice (Figure 8A). While kanamycin or HY8002 treatment did not cause a significant change in the mRNA levels of OCLN, these levels were higher in the HY8002 treatment groups than those in the control group (Figure 8B). In addition, we observed that the mRNA levels of TLR2 and TLR4 in the mouse small intestine were significantly decreased upon kanamycin treatment. However, HY8002 administration restored the mRNA levels of TLR2 but not those of TLR4 (Figures 8B,C). Thus, we inferred that HY8002 has a positive effect on the expression of tight junction-related genes in the intestinal epithelial cells by particularly increasing the gene expression of *ZO-1*, related to TLR2. When TLR2 signaling was blocked with a blocking antibody, IgA secretion in Peyer’s patch cells and *ZO-1* expression in intestinal epithelial cells by HY8002 were significantly reduced. These results demonstrates that HY8002 enhances the intestinal immune response and tight junctions through TLR2 signaling (Figures 10A,B). This observation concurred with that of a recent study wherein a specific strain of *Bifidobacterium bifidum* relieved dextran sulfate sodium-induced colitis by strengthening the tight junction of intestinal epithelial cells through TLR2 signaling (Al-Sadi et al., 2021).

The microbiome plays a pivotal role in the development and maintenance of the mammalian immune system. The GIT has a total area of 400 m^2^ and along with an epithelial barrier forms a part of the human body that is greatly affected by the microbiome. It has a complex, open, and integrated ecology that is the most exposed to the external environment (Lazar et al., 2018). Therefore, the intestinal microbiome regulates the intestinal immune response and tight junctions either directly or indirectly. Studies have reported that the strengthening effect that probiotics and prebiotics have on the intestinal mucosa is because of changes in the intestinal microbiome (Duranti et al., 2016; Shi et al., 2020). However, some conflicting reports claim that that the effects some probiotics have on relieving intestinal mucosal inflammation are not correlated with changes in the intestinal microbiome (Ojima et al., 2020). In this study, we demonstrated that in addition to significantly reducing the α-diversity of the mouse gut microbiota, antibiotics also altered the proportions of several microbial genera in the intestine. Notably, HY8002 reversed some of these microbiome changes (Figures 9B–D); it restored the proportion of *Oscillibacter* genus that was sharply decreased by kanamycin treatment. In contrast, the intestinal abundances of the *Clostridium* and *Blautia* genera were greatly increased by kanamycin and decreased by HY8002. Recently, studies have reported that *Oscillibacter* are reduced in gastric cancer and Crohn’s disease, whereas *Blautia* are associated with diarrhea and rheumatoid arthritis (Zhai et al., 2019; Ojima et al., 2020). The bacterial genus *Clostridium* includes multiple pathogenic strains and is well associated with enteritis and antibiotic-induced diarrhea (Rea et al., 2011; Dzunkova et al., 2016; Shao et al., 2020). We found that the *Oscillibacter* genus was positively correlated with intestinal IgA secretion and ZO-1 and TLR2 levels. In contrast, the *Clostridium* and *Blautia* genera had a negative correlation with IgA secretion and ZO-1 and TLR2 levels (Figure 9E). The correlation between the abundance of *Oscillibacter* and *Blautia* and intestinal TLR2 levels was first revealed in our study. From these results, it can be inferred that HY8002 itself not only strengthens the immune response and tight junction of the intestinal mucosa through TLR2 signaling, but also improves intestinal integrity by affecting the intestinal microbiome. Therefore, we confirmed that the bifidobacterial strain HY8002 exhibits excellent abilities to enhance intestinal immunity and strengthen the intestinal barrier. Moreover, it can improve the intestinal microbiota composition and has a high potential as a probiotic. Currently, we are planning a clinical trial to validate the effect of HY8002 on intestinal and systemic immune imbalance. Nevertheless, the limitation of this study is that the secretion of antimicrobial peptides, such as Reg3b and defensins that affects the homeostasis of the intestinal microbiota and the effect of HY8002 on individual immune cells present in Peyer’s patch were not confirmed. Therefore, further studies are needed to investigate the effect of HY8002 on the activation of immune cells, such as T cells and B cells, in MALT and the secretion of antimicrobial peptides from the intestinal epithelium.

## Conclusion

This study validated the potential of *Bifidobacterium animalis* ssp. *lactis* HY8002 as a probiotic to help maintain intestinal integrity. *In vitro*, it displayed a positive tolerance toward simulated digestive tract conditions, adhered well with the intestinal epithelium, and induced high IgA secretion by Peyer’s patch cells. *In vivo*, it restored tight junctions, enhanced systemic and intestinal IgA secretion, and improved the intestinal microbiota balance. In addition, HY8002 was genetically verified as a non-virulent or a safe strain. These results indicate that HY8002 can be used as a beneficial probiotic strain in animals and humans because of its protective effect on intestinal integrity. It is evident that HY8002 is greatly promising for the maintenance of a healthy physiological state, as it enhances the immunity and adhesion margins of the intestinal mucosa. However, further studies are needed to elucidate the molecular communication mechanisms between HY8002 and the host immune system and their effects on the gut microbiome.

## Data Availability Statement

The datasets presented in this study can be found in online repositories. The names of the repository/repositories and accession number(s) can be found in the article/Supplementary Material.

## Ethics Statement

The animal study was reviewed and approved by the Institutional Animal Care and Use Committee of hy Co., Ltd. (approval number: AEC-2021-00008-Y).

## Author Contributions

JK, KH, and J-LL contributed to conception and design of the study. JK and S-JB organized the database. J-YK performed the statistical analysis. JK wrote the first draft of the manuscript. JK, EC, and J-YK wrote sections of the manuscript. All authors contributed to the article and approved the submitted version.

## Conflict of Interest

JK, S-JB, J-YK, EC, KH, J-JS, and J-LL are employed by hy Company Limited (hy Co., Ltd.).

## Publisher’s Note

All claims expressed in this article are solely those of the authors and do not necessarily represent those of their affiliated organizations, or those of the publisher, the editors and the reviewers. Any product that may be evaluated in this article, or claim that may be made by its manufacturer, is not guaranteed or endorsed by the publisher.

## Footnotes

^1^ http://huttenhower.sph.harvard.edu/galaxy
