# Supplementary material for: The Probiotic Strain Bifidobacterium animalis ssp. lactis HY8002 Potentially Improves the Mucosal Integrity of an Altered Intestinal Microbial Environment
Source: Front Microbiol. 2022 Apr 29;13:817591. doi: 10.3389/fmicb.2022.817591 (PMC9102380; doi:10.3389/fmicb.2022.817591)
Supplement: Supplementary file 1 [file Table_1.DOCX]

Supplementary Material

## Supplementary Figures


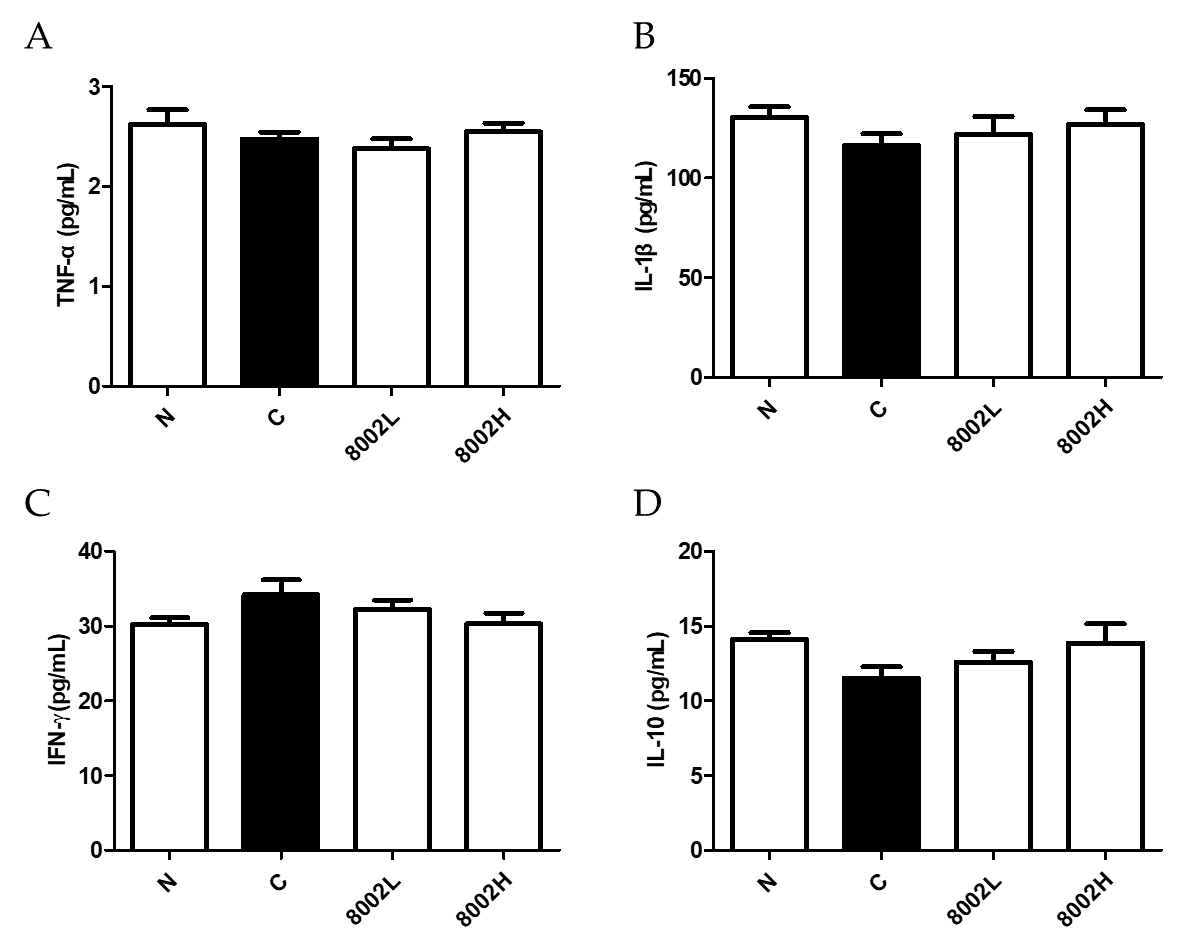


**Supplementary Figure 1.** Effect of HY8002 administration on plasma (A) TNF-α, (B) IL-1β, (C) IFN-γ, and (D) IL-10 in kanamycin-treated mice. The results are expressed as the means ± SEM. N, normal group; C, kanamycin administration group; 8002L, kanamycin with 1.0х10^8^ CFU/kg/day HY8002 administration group; 8002H, kanamycin with 1.0х10^9^ CFU/kg/day HY8002 administration group.


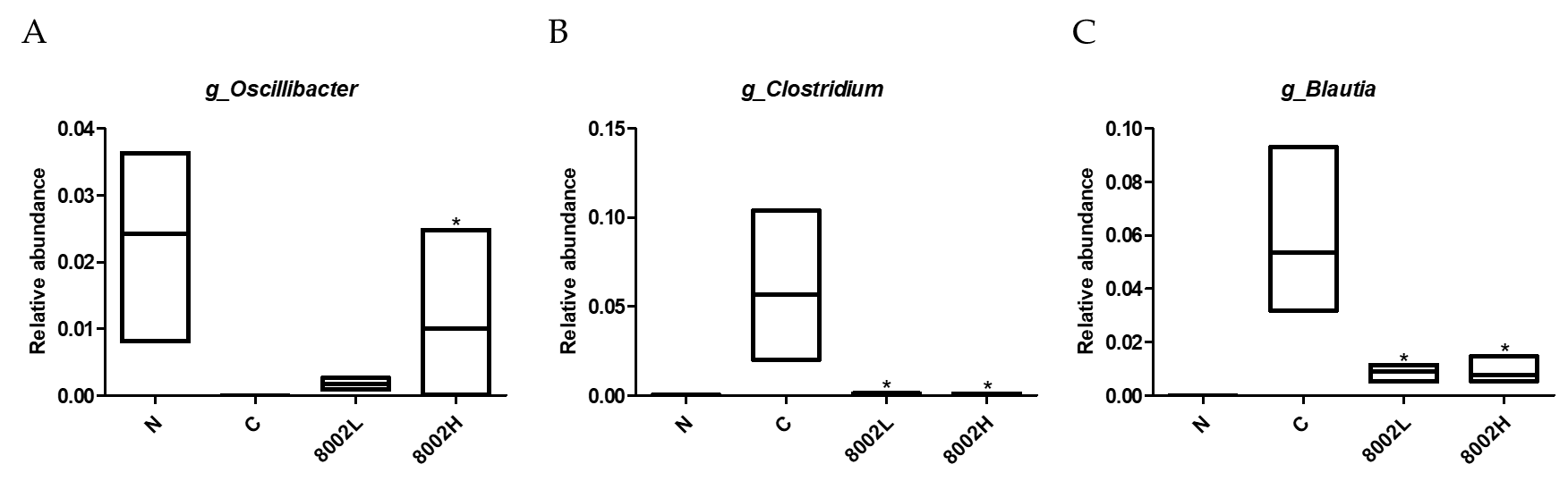


**Supplementary Figure 2.** Three taxa that showed significant recovery in microbial abundance after HY8002 treatment were selected and presented as box plots. The data are presented as minimum-to-maximum values. * *p* < 0.05 compared with kanamycin-treated control group.


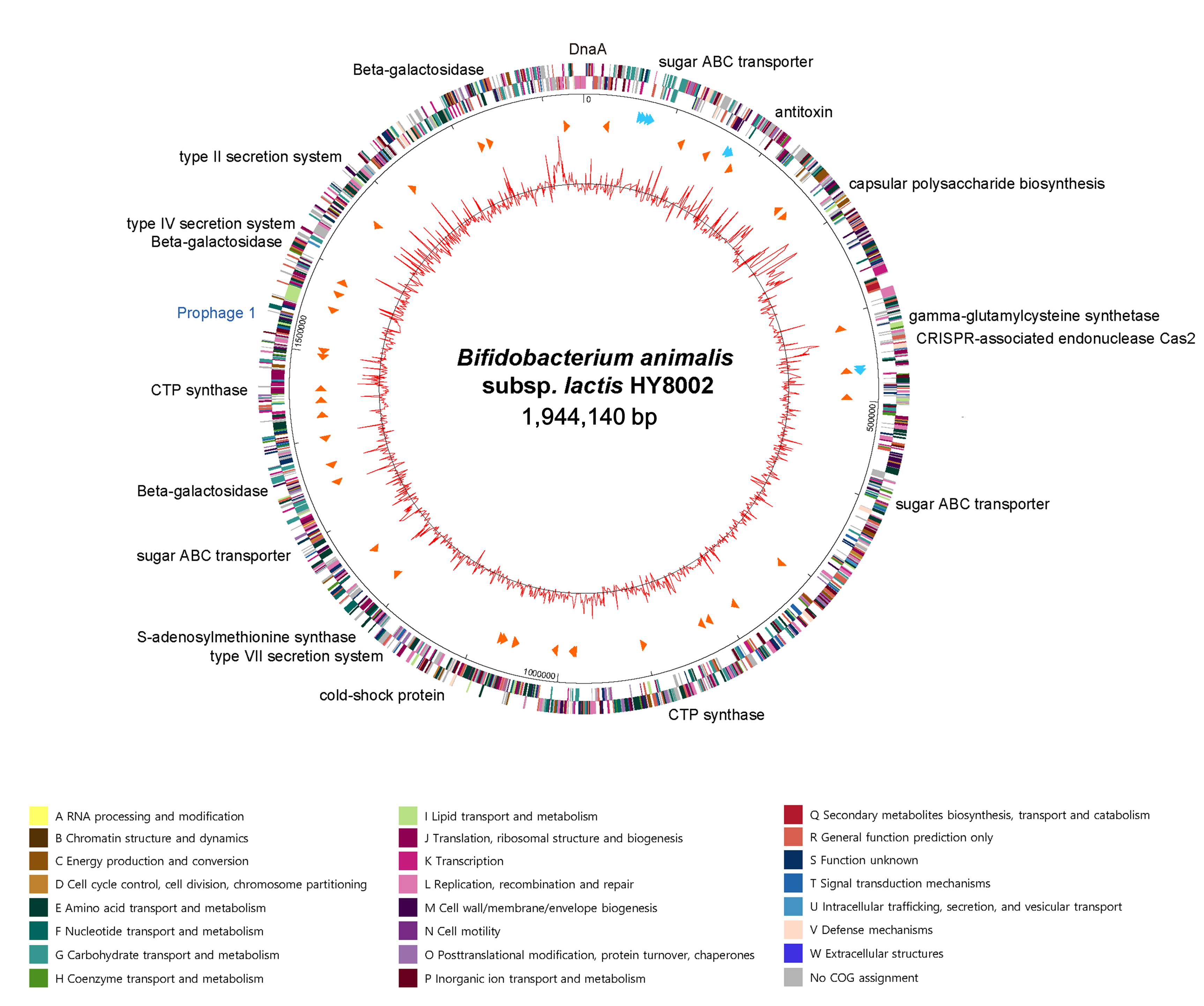


**Supplementary Figure 3.** Genomic map of the HY8002 whole-genome.


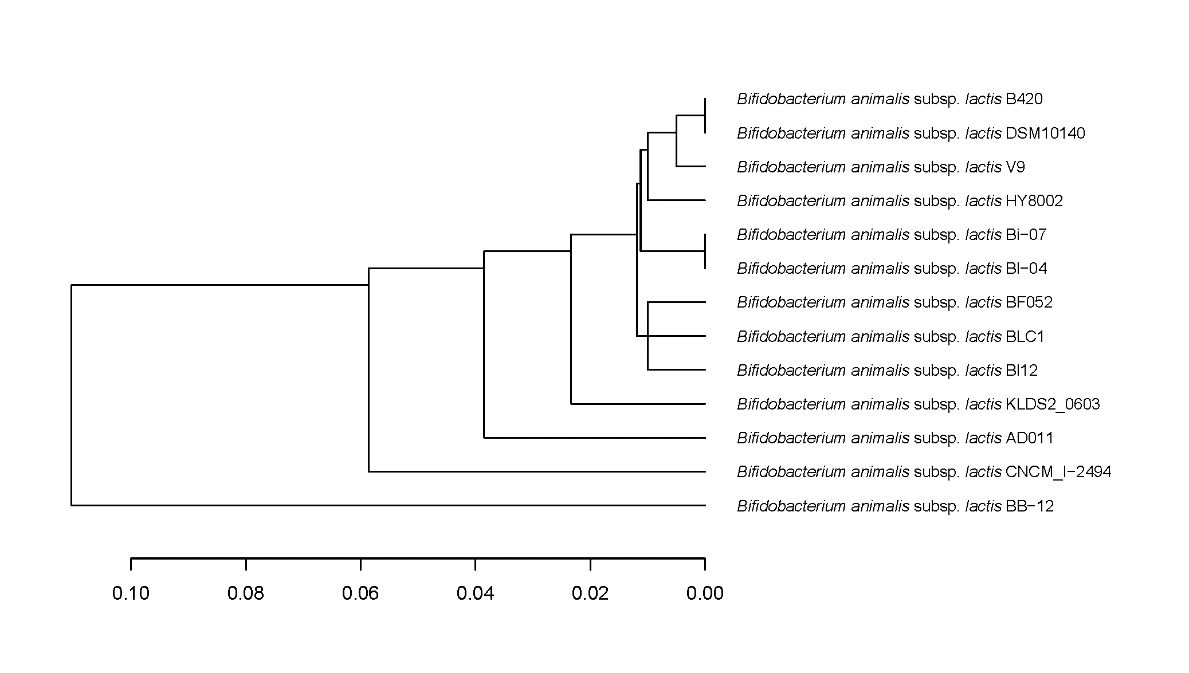
**Supplementary Figure 4.** Phylogenetic tree of HY8002 based on whole-genome sequence.
